# Early versus late termination for fetal anomalies: Women's perspectives and psychological impact in a mixed methods study

**DOI:** 10.1111/aogs.70122

**Published:** 2026-01-09

**Authors:** Eline E. R. Lust, Kim Bronsgeest, Lidewij Henneman, Neeltje M. T. H. Crombag, Caterina M. Bilardo, Robert‐Jan H. Galjaard, Esther Sikkel, Audrey B. C. Coumans, Ayten Elvan‐Taşpınar, Sander Galjaard, Attie T. J. I. Go, Gwendolyn T. R. Manten, Eva Pajkrt, Elisabeth van Leeuwen, Monique C. Haak, Mireille N. Bekker

**Affiliations:** ^1^ Department of Obstetrics and Gynecology University Medical Center Utrecht Utrecht The Netherlands; ^2^ Department of Obstetrics and Gynecology Leiden University Medical Center Leiden The Netherlands; ^3^ Department of Human Genetics and Amsterdam Reproduction and Development Research Institute Amsterdam UMC, Location Vrije Universiteit Amsterdam Amsterdam The Netherlands; ^4^ Department of Obstetrics and Gynecology Amsterdam UMC Amsterdam the Netherlands; ^5^ Department of Clinical Genetics Erasmus University Medical Center Rotterdam The Netherlands; ^6^ Department of Obstetrics and Gynecology Radboud University Medical Center Nijmegen The Netherlands; ^7^ Department of Obstetrics and Gynaecology Maastricht UMC+ Maastricht The Netherlands; ^8^ GROW‐Research Institute for Oncology and Reproduction Maastricht The Netherlands; ^9^ Department of Obstetrics and Gynecology University Medical Center Groningen, University of Groningen Groningen The Netherlands; ^10^ Department of Obstetrics and Gynecology Erasmus University Medical Center Rotterdam The Netherlands; ^11^ Department of Obstetrics and Gynecology Isala, Zwolle The Netherlands

**Keywords:** abnormalities, abortion, anxiety, early diagnosis, fetal screening, fetus, induced, pregnancy complications, prenatal ultrasonography, psychology, surveys and questionnaires

## Abstract

**Introduction:**

A frequently cited benefit of the first‐trimester anomaly scan (FTAS) is that it reduces psychological impact by enabling earlier termination of pregnancy (TOP). However, the impact of early versus late TOP due to fetal anomalies remains unclear. This study evaluates the psychological impact and perspectives associated with early versus late TOP.

**Material and Methods:**

A prospective mixed methods study was conducted. The early group (TOP <18 weeks) included women with an abnormal FTAS; the late group (TOP 20–24 weeks) included women with an abnormal second‐trimester scan (SAS), abnormal FTAS, or normal FTAS followed by abnormal SAS. Women completed questionnaires 2 (T1) and 6 months (T2) postpartum addressing psychological impact using validated scales (State–Trait Anxiety Inventory, Edinburgh Depression Scale, Impact of Event Scale, Perinatal Grief Scale) and study‐specific questions. Semi‐structured interviews were conducted with women and their partners 3–6 months after termination.

**Results:**

149 women with early TOP (15 + 2 weeks, range 14 + 4–16 + 1) and 129 with late TOP (22 + 0, 21 + 0–23 + 1) completed T1. In both groups, the majority had clinically relevant anxiety at T1 and T2 and moderate/severe distress at T1. The late TOP group had higher median depression and mean grief scores at T1 (5.0, range 3.0–8.0 vs. 4.0, range 2.0–7.0, *p* = 0.004) (85.9 ± 21.0 vs. 76.5 ± 22.4, *p* < 0.001) and at T2 (4.0, 1.0–7.0 vs. 3.0, 1.0–6.0, *p* = 0.043) (81.3 ± 22.9 vs. 70.8 ± 22.6, p < 0.001), respectively, and higher mean distress scores at T1 (33.8 ± 13.3 vs. 30.2 ± 14.7, *p* = 0.034). Of 51 interviews with women and partners (22 early, 29 late TOP), four themes were identified: fetal attachment, time pressure, grief, and reflections on gestational age. Most late TOP participants expressed strong fetal attachment; for early TOP participants, the experiences were more variable. Half of the late TOP participants reported time pressure due to the legal limit. Perceived grief and impact were substantial in both groups.

**Conclusions:**

Our findings suggest that early TOP is associated with lower psychological impact compared to late TOP, mainly in the first months postpartum. This may reflect less intense fetal attachment and more time for reproductive decision‐making for some parents, supporting the presumed benefit of earlier intervention. Nevertheless, TOP causes a significant emotional impact at any gestational age.

## Graphical abstract

This mixed method study evaluated the psychological impact and perspectives associated with early and late termination of pregnancy (TOP). Early TOP due to fetal anomalies is associated with a lower psychological impact compared to late TOP. Pregnancy termination has a substantial emotional impact regardless of gestational age.

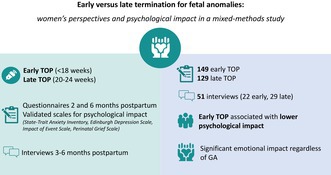

**Abbreviations**
FTAS: first‐trimester anomaly scan
TOP: termination of pregnancy

**Key message**

Early pregnancy termination following an abnormal first‐trimester anomaly scan is associated with lower psychological impact compared to late termination, possibly due to less intense fetal attachment and more time for decision‐making. Nevertheless, pregnancy termination carries a significant emotional impact.

## 1 INTRODUCTION

Ultrasound enables timely diagnosis of fetal anomalies, providing parents essential information for reproductive decision‐making. In many countries, termination of pregnancy (TOP) for fetal anomalies is legally and morally accepted as a reproductive option, though specific laws, regulations, and procedures vary.^1^ Advancements in ultrasound technology have significantly improved early detection of structural anomalies, with increasing evidence suggesting many anomalies detected during second‐trimester ultrasound screening can now be identified by a first‐trimester anomaly scan (FTAS).^2^, ^3^

The Netherlands introduced the FTAS as part of an implementation study into its national prenatal screening program in September 2021, aiming to detect major congenital anomalies.^3^ A commonly cited benefit of the FTAS is that it provides parents with additional time for reproductive decision‐making and, if preferred, the option of an earlier TOP in case of fetal anomalies.^3^ It should be noted that establishing a diagnosis in early pregnancy can be challenging and repeated examinations may be necessary, sometimes leading to a prolonged and stressful waiting period for diagnosis and decisions regarding termination, even though technology has improved early detection of structural anomalies.

The psychological impact of TOP for fetal anomalies can be significant, affecting both women and their partners.^4^, ^5^, ^6^, ^7^, ^8^, ^9^, ^10^, ^11^, ^12^, ^13^ Studies suggest that earlier gestational age at termination may be associated with less psychological distress, including lower levels of grief and posttraumatic stress.^4^, ^7^, ^8^ However, the publication dates of almost all of these studies indicate that research on the psychological impact of pregnancy termination has not kept pace with the rapid advancements in ultrasound technology and screening methods—highlighting a gap in the current body of knowledge. In particular, little is known about the psychological impact of TOP following ultrasound screening for structural anomalies. Most studies were published before 2009 or do not differentiate between early and late TOP, nor account for the pre‐test risk of anomalies prior to the abnormal result.^4^, ^5^, ^6^, ^7^, ^8^, ^9^, ^10^, ^11^, ^12^, ^13^

Therefore, we conducted a mixed methods study evaluating the psychological impact and perspectives of early versus late TOP following the diagnosis of a fetal anomaly detected by ultrasound in the screening setting. Using a large cohort, our findings provide crucial insight into the influence of gestational age on psychological outcomes after TOP and its implications for prenatal care. This mixed methods approach allows for both the quantification of the psychological impact of TOP and a deeper understanding of the underlying factors contributing to these outcomes.

## 2 MATERIAL AND METHODS

### 2.1 Design

A prospective concurrent mixed methods study design was employed, combining questionnaire data with semi‐structured individual interviews. Data were collected simultaneously, analyzed separately, and compared to gain a comprehensive understanding of the experiences with early and late TOP due to fetal anomalies.

### 2.2 Setting

This study was part of the nationwide “IMplementation of fIrst Trimester Anomaly Scan (IMITAS) study,” assessing the introduction of the FTAS into the Dutch prenatal screening program. The FTAS was performed between 12 + 3 and 14 + 3 weeks of gestation.^3^ A dating scan between 10 and 12 + 6 weeks gestation was required prior to the FTAS.^14^ The screening program further includes the second‐trimester anomaly scan (SAS) at 18–21 weeks gestation and noninvasive prenatal testing (NIPT).^3^, ^15^ TOP is legally permitted up to 24 completed weeks of gestation in the Netherlands.

Part of this study was conducted before FTAS availability in the Netherlands, whereas the other part was conducted after FTAS introduction (September 2021). Late termination was defined as termination between 20 and 24 weeks of gestation; termination before 18 weeks was considered early. To ensure a clear distinction between early and late TOP based on gestational age, terminations performed between 18 and 20 weeks were excluded. The late group included women who terminated after an abnormal SAS, after a normal FTAS followed by an abnormal SAS, or after an abnormal FTAS but who decided to terminate after 20 weeks of gestation (e.g., because they were waiting for additional results before making a decision). The early group terminated following an abnormal FTAS.

Ethical approval was granted by the Dutch Ministry of Health, Welfare, and Sport (license 3 219 987‐1 012 059‐PG) and based on positive advice by the Health Council of the Netherlands (No. 2021/30, 30 June 2021). The phase before FTAS introduction was approved by the Medical Ethical Review Committee Utrecht (No. 20/765, December 1, 2020). Written informed consent was obtained from all participants.

### 2.3 Participants and data collection

Questionnaires were distributed between January 2021 and May 2023. Before FTAS introduction, women were recruited across all eight Dutch tertiary care centers via two routes. First, women were invited to participate by their obstetrician or social worker at the outpatient clinic. Second, recruitment occurred through an ongoing IMITAS survey study for women with an abnormal SAS. If their responses indicated that the pregnancy had been terminated due to fetal anomalies, these women were asked to provide consent to participate in the current study.

In the phase following FTAS introduction, the national prenatal registration database (Peridos) was used to identify women with an abnormal FTAS or SAS. A random sample of these women was invited to complete the questionnaire. If pregnancy termination had occurred, women were asked to provide consent to participate in this study on TOP.

Women received questionnaires by email at 2 (T1) and 6 months (T2) after delivery. Exclusion criteria for the questionnaire study included insufficient Dutch proficiency and TOP due to abnormal NIPT or abnormal detailed diagnostic scans because of a high prior risk for fetal anomalies.

### 2.4 Psychometric questionnaires

The multidisciplinary research team developed the questionnaire. Both validated scales as well as specially designed questions for this study were used. Key topics included anxiety, depression, distress, grief, resumption of work, and perceived time–pressure.

Anxiety was measured by the validated Dutch 6‐item short version of the Spielberger State–Trait Anxiety Inventory (STAI).^16^, ^17^ Total scores ranged from 20 to 80, with higher scores reflecting more anxiety. A total score of ≥40 was considered a clinically significant level of anxiety.^18^

Depression was assessed using the validated 5‐item short version of the Edinburgh Depression Scale (EDS).^19^, ^20^ Total scores ranged from 0 to 15, with values ≥7 reflecting a moderate‐symptom level.^19^

Distress from TOP was measured using a Dutch version of the Impact of Event Scale (IES) consisting of 15 items.^21^, ^22^ Total scores ranged from 0 to 75, with higher scores reflecting more subjective stress. The cutoff point is 26, with 26–43 points reflecting moderate impact and ≥44 points indicating severe impact.^23^

Grief was measured with the 33‐item translated short version of the Perinatal Grief Scale (PGS).^24^ Total score ranged from 33 to 165, with scores ≥91 considered worrisome, indicating a higher probability of greater vulnerability to the loss.^25^

A subanalysis addressed heterogeneity within the late TOP group. Sociodemographic variables included maternal age, gestational age at termination, living children, education, ethnicity, religion, conception method, health perception, reason for TOP, and time since delivery.

Descriptive statistics were used to summarize baseline characteristics and outcomes. Categorical variables were compared using chi‐squared tests, continuous variables using unpaired *t*‐tests, and nonnormally distributed data using Mann–Whitney U tests. Statistical significance was set at *p* < 0.05. Analyses were performed using SPSS version 29.

### 2.5 Interviews

Interviews were conducted between July 2021 and October 2022. Women and their partners were recruited in two ways. First, through the questionnaire used in this study and second via three tertiary care centers (Leiden University Medical Centre, University Medical Center Utrecht, University Medical Center Groningen), where they were invited by an obstetrician or social worker. Maximum variation in the sample was aimed for, considering ethnicity and educational levels. Recruitment continued until data saturation. Participants had to speak Dutch or English and could participate individually or with their partner. Women and partners were excluded if TOP followed abnormal NIPT or an abnormal detailed diagnostic scan due to high prior risk of fetal anomalies.

Participants were interviewed 3–6 months after TOP by two researchers (EERL and KB) via Microsoft Teams due to COVID‐19. A structured guide, developed by the multidisciplinary IMITAS team, covered decision‐making for TOP, perception of the future child (attachment, parental identity), mental well‐being, grief, coping, and support (Appendix S1). Interviews (40–120 min) were audio and video‐recorded, then transcribed verbatim. Data were analyzed using the Qualitative Analysis Guide of Leuven (QUAGOL), an iterative process involving repeated reading, coding, and team discussions,^26^ which aligns with thematic content analysis. Atlas.ti Web was used for coding, performed independently by EERL and KB, with consensus reached through discussion. Themes were derived from codes and categorized into main and subtopics. Supporting quotes were translated from Dutch, referenced in‐text (e.g., Quote 1), and identified by respondent (R) number, group (early or late TOP), and mother or partner.

### 2.6 Classification of fetal anomalies

Fetal diagnoses were either self‐reported by participants or extracted from the electronic patient file, depending on the recruitment method. They were classified by two of the authors (EERL, MNB) based on Kaasen et al.^27^ This classification was applied to allow comparison of the types of fetal anomalies between the early and late TOP group. In case of insufficient information about the fetal diagnosis, severity was categorized as “severity not classified; anomaly awaiting clarification.” The categories are presented in Table 1.^27^

**TABLE 1:** Classification of fetal anomalies.

Category	Definition
Category 1	Lethal or serious anomaly with no available treatment (e.g., acrania, skeletal dysplasia with small thorax, holoprosencephaly, bilateral renal agenesis)
Category 2	Serious anomaly with available treatment, but with prognostic ambiguity (e.g., myelomeningocele with hydrocephalus, diaphragmatic hernia with lung: head ratio <1.0, hypoplastic left heart syndrome)
Category 3	Anomaly with mild to moderate severity, with available treatment, usually giving good results, but with prognostic ambiguity (e.g., bilateral clubfoot or cleft lip with no other markers, but condition known to be associated with syndromes not apparent prenatally)
Category 4	Anomaly with mild to moderate severity, with available treatment, usually giving good results, and without prognostic ambiguity (e.g., gastroschisis, unilateral renal cysts, unilateral clubfoot)
Category 5	Severity not classified; anomaly awaiting clarification or missing data on diagnosis. This definition was slightly adapted from the original classification

*Note*: Classification based on Kaasen et al.^27^

## 3 RESULTS

### 3.1 Questionnaire

In total, 350 women received a questionnaire at T1, of which 79.4% (*n* = 278) were completed and used for analysis. T2 was completed by 91.4% (254/278). Baseline characteristics are shown in Table 2. The late termination group consisted of 58.1% (*n* = 75) with an abnormal SAS but normal FTAS, 26.4% (*n* = 34) with an abnormal SAS and no FTAS, and 15.5% (*n* = 20) with an abnormal FTAS but with a TOP after 20 weeks of gestation. In the early TOP group, 48.3% (*n* = 72) of fetal anomalies were classified as Category 1, 45.6% (*n* = 68) as Category 2, 1.3% (*n* = 2) as Category 3, 0.7% (*n* = 1) as Category 4, and 4.0% (*n* = 6) as Category 5. In the late TOP group, the distribution was 18.6% (*n* = 24), 71.3% (*n* = 92), 4.7% (*n* = 6), 1.6% (*n* = 2), and 3.9% (*n* = 5), respectively.

**TABLE 2:** Baseline characteristics of questionnaire respondents (*n* = 278).

Characteristics	Early TOP (*n* = 149)	Late TOP (*n* = 129)	*p*
Maternal age, years (mean ± SD)	32.3 ± 3.9	31.5 ± 3.8	0.102
GA at TOP, weeks (median, IQR)	15 + 2 (14 + 4–16 + 1)	22 + 0 (21 + 0–23 + 1)	<0.001
Living children at first questionnaire, *n* (%)			0.683
0	76 (51.0)	68 (52.7)	0.776
≥1	73 (49.0)	61 (47.3)	
Education level, *n* (%)^a^			0.118
Low	8 (5.4)	4 (3.1)	
Intermediate	33 (22.1)	42 (32.6)	
High	108 (72.5)	83 (64.3)	
Ethnicity^b^			0.673
Dutch	131 (87.9)	108 (84.4)	
Other Western	11 (7.4)	13 (10.2)	
Non‐Western	7 (4.7)	7 (5.5)	
Religious affiliation, *n* (%)^c^			0.624
Not religious	112 (77.2)	102 (79.7)	
Religious	33 (22.8)	26 (20.3)	
Method of conception			0.334
Natural	131 (87.9)	118 (91.5)	
Assisted	18 (12.1)	11 (8.5)	
Health perception			
Good—very good—excellent	144 (96.6)	127 (98.4)	0.338
Moderate—poor	5 (3.4)	2 (1.6)	
Time since delivery at T1, weeks (median, IQR)	8 + 2 (8 + 0–9 + 1)	8 + 1 (8 + 0–9 + 0)	0.089
Time since delivery at T2, weeks (median, IQR)^d^	24 + 1 (24 + 0–25 + 1)	24 + 1 (24 + 0–25 + 0)	0.537

Abbreviations: FTAS, first‐trimester anomaly scan; GA, gestational age; IQR, interquartile range; SAS, second‐trimester anomaly scan; SD, standard deviation; TOP, termination of pregnancy.

^a^ Education level: Level categorized as low (no education or highest attained educational level primary school, low level of secondary school, lower vocational training), intermediate (middle vocational training or high level of secondary school), or high (higher vocational training or university/doctorate).

^b^ Ethnicity: Categorized as Dutch: both parents were born in the Netherlands; other Western: one or both parents were born in Europe (excluding Turkey), North America, Oceania, Indonesia, or Japan; non‐Western: one or both parents were born in Africa, Latin America, Asia (excluding Indonesia or Japan), or Turkey. Maternal country of birth was leading if both parents were born abroad. 1 missing value late TOP.

^c^ Religious affiliation: 4 missing values early TOP, 1 missing value late TOP.

^d^ Time since delivery at T2: 8 missing values early TOP, 16 missing values late TOP.

### 3.2 Anxiety

Respondents in the late termination group did not differ significantly in mean total STAI scores compared to those in the early termination group at both time points (Table 3).

**TABLE 3:** Psychometric outcomes for women with an early and late TOP.

Outcome measures	Early TOP^a^ (*n* = 149)	Late TOP^b^ (*n* = 129)	*p*
Anxiety (STAI)			
2 months postpartum (T1)			
Mean ± SD	43.3 ± 13.6	46.1 ± 11.4	0.067
Clinically significant score (≥40, *n* (%))	92.0 (61.7)	94.0 (72.9)	0.049
6 months postpartum (T2)			
Mean ± SD	41.4 ± 12.8	43.3 ± 11.6	0.231
Clinically significant score (≥40, *n* (%))	78 (55.3)	72 (63.7)	0.176
Depression (EDS)			
2 months postpartum (T1)			
Median IQR	4.0 (2.0–7.0)	5.0 (3.0–8.0)	0.004
Moderate symptom level (≥7, *n* (%))	41.0 (27.5)	49.0 (38.0)	0.063
6 months postpartum (T2)			
Median IQR	3.0 (1.0–6.0)	4.0 (1.0–7.0)	0.043
Moderate symptom level (≥7, *n* (%))	23.0 (16.3)	30.0 (26.5)	0.046
Post‐traumatic stress (IES)			
2 months postpartum (T1)			
Mean ± SD	30.2 ± 14.7	33.8 ± 13.3	0.034
Clinically significant score (≥26, *n* (%))	87 (58.4)	93 (72.1)	0.017
Moderate impact (26–43, *n* (%))	54 (36.2)	66 (51.2)	0.012
Severe impact (≥44, *n* (%))	33 (22.1)	27 (20.9)	0.806
6 months postpartum (T2)			
Mean ± SD	26.7 ± 15.5	29.4 ± 13.3	0.134
Clinically significant score (≥26, *n* (%))	65 (46.1)	70 (61.9)	0.012
Moderate impact (26–43, *n* (%))	41 (29.1)	52 (46.0)	0.005
Severe impact (≥44, *n* (%))	24 (17.0)	18 (15.9)	0.816
Grief (PGS)			
2 months postpartum (T1)			
Mean ± SD	76.5 ± 22.4	85.9 ± 21.0	<0.001
Clinically significant score (≥91, *n* (%))	36 (24.2)	52 (40.3)	<0.001
6 months postpartum (T2)			
Mean ± SD	70.8 ± 22.6	81.3 ± 22.9	<0.001
Clinically significant score (≥91, *n* (%))	24 (17.0)	38 (33.6)	0.002

Abbreviations: EDS, Edinburgh Depression Scale; IES, Impact of Event Scale; PGS, Perinatal Grief Scale; SD, Standard deviation; STAI, State–Trait Anxiety Inventory; TOP, Termination of pregnancy.

^a^ Six months postpartum, early TOP *n* = 141.

^b^ Six months postpartum, late TOP *n* = 113.

Most women in both groups had clinically relevant anxiety scores (STAI score ≥40) at T1 and T2 (Table 3). However, a significantly larger proportion of women in the late TOP group had a clinically significant level of anxiety at T1 (72.9% versus 61.7%, *p = 0.049*).

### 3.3 Depression

Respondents in the late termination group had a significantly higher median total EDS score compared to those in the early termination group at both T1 (5.0, IQR 3.0–8.0 vs 4.0, IQR 2.0–7.0, *p =* 0.004, Table 3) and T2 (4.0, IQR 1.0–7.0 vs. 3.0, IQR 1.0–6.0, *p* = 0.043).

The proportion of women with a moderate symptom level (EDS score ≥7) was not significantly different between both groups at T1 but was significantly higher in the late termination group at T2 (26.5% vs. 16.3%, *p* = 0.046, Table 3).

### 3.4 Distress

Respondents in the late termination group had a significantly higher mean total IES score at T1 compared to respondents with an early termination (33.8 ± 13.3 vs. 30.2 ± 14.7, *p* = 0.034, Table 3). This difference did not persist at a statistically significant level at T2.

Most women in both groups had moderate or severe impact levels (IES score ≥26) at T1, with a significantly higher proportion in the late termination group (72.1% vs. 58.4%, *p* = 0.017, Table 3). At T2, the proportion remained significantly higher in the late termination group compared to the early termination group (61.9% vs. 46.1%, *p* = 0.012). Further analysis showed no significant difference in the proportion of women with severe impact (IES score ≥44) at both time points (Table 3).

### 3.5 Grief

Respondents in the late termination group had a significantly higher total mean PGS score compared to those in the early termination group at both T1 (85.9 ± 21.0 vs. 76.5 ± 22.4, *p* < 0.001, Table 3) and T2 (81.3 ± 22.9 vs. 70.8 ± 22.6, *p* < 0.001).

A significantly larger proportion of women in the late termination group experienced a high degree of grief at T1 (40.3% vs. 24.4%, *p* < 0.001, Table 3) compared to the early termination group. This difference remained significant at T2 (33.6% vs. 17.0%, *p* = 0.002).

### 3.6 Resumption of work

Most women in both groups had resumed work at T2, 6 months after the date of delivery (92.7% in the late termination group versus 95.6% in the early termination group, *p* = 0.336). The median time to work resumption after TOP was significantly longer in the late TOP group (51 days IQR 32–83 days) compared to the early TOP group (37 days IQR 17–58 days, *p* < 0.001).

### 3.7 Perceived time pressure

At T1, 22.5% (*n* = 29) of women in the late TOP group (strongly) agreed that they would have appreciated having more time for decision‐making concerning pregnancy termination, compared to 2.7% (*n* = 4) in the early TOP group.

Subanalysis of the psychometric outcomes within the late TOP group (Table S1), comparing women with an abnormal FTAS, a normal FTAS followed by an abnormal SAS, or an abnormal SAS, showed no significant differences between these subgroups.

### 3.8 Interviews

In total, 51 interviews were conducted with 50 women and 13 partners. Of these, 22 interviews involved participants with an early pregnancy TOP and 29 with a late TOP. In the late TOP group, 48.3% (*n* = 14) of the interviews were with participants who had an abnormal SAS and no FTAS, 41.4% (*n* = 12) with an abnormal SAS but a normal FTAS, and 10.3% (*n* = 3) with an abnormal FTAS. Participant characteristics are shown in Table 4. In the early TOP group, 54.5% (*n* = 12) of fetal anomalies were classified as Category 1, 40.9% (*n* = 9) as Category 2, and 4.5% (*n* = 1) as Category 4. In the late TOP group, the distribution was 27.6% (*n* = 8) for Category 1, 69.0% (*n* = 20) for Category 2, and 3.4% (*n* = 1) for Category 3.

**TABLE 4:** Characteristics of interviewed women (*n* = 50) and partners (*n* = 13)^a^.

Characteristics	
Age, years (mean ± SD)	
Women who underwent TOP	32.7 ± 3.9
Partners	34.7 ± 4.8
Education level, *n* (%)^b^	
Low	0 (0.0)
Intermediate	10 (16.9)
High	49 (83.1)
Ethnicity of women who underwent TOP^c^	
Dutch	44 (88.0)
Other Western	5 (10.0)
Non‐Western	1 (2.0)
Women who underwent TOP having living children, *n* (%)	
Yes	21 (42.0)
No	29 (58.0)
Mean GA at TOP, weeks (range)	
Early TOP	15 + 3 (13 + 6–17 + 6)
Late TOP	22 + 3 (20 + 4–23 + 6)

Abbreviations: GA, Gestational Age; SD, Standard deviation; TOP, Termination of pregnancy.

^a^ 63 individuals comprising 12 couples.

^b^ Education level: Level categorized as low (no education or highest attained educational level primary school, low level of secondary school, lower vocational training), intermediate (middle vocational training or high level of secondary school), or high (higher vocational training or university/doctorate). 4 missing values.

^c^ Ethnicity: Categorized as Dutch: both parents were born in the Netherlands; other Western: one or both parents were born in Europe (excluding Turkey), North America, Oceania, Indonesia, or Japan; non‐Western: one or both parents were born in Africa, Latin America, Asia (excluding Indonesia or Japan), or Turkey. Maternal country of birth was leading if both parents were born abroad.

### 3.9 Themes

Perspectives identified from interviews were allocated to 4 overarching themes: (1) fetal attachment with three subcategories (during pregnancy, after the abnormal scan, parental identity after birth), (2) time pressure, (3) grief, (4) reflections on gestational age (Table 5). These themes are described below in detail with illustrative quotes (Table 6).

**TABLE 5:** Overarching themes with definitions.

Theme	Definition
Fetal attachment	The perceived emotional bond participants have experienced with their fetus as well as the development of a sense of parental identity
During pregnancy	
After the abnormal scan	
Parental identity after birth	
Time pressure	Perceived pressure associated with the legal limit for termination in the Netherlands
Grief	Ongoing emotional impact following the termination
Reflections on gestational age	Influence of early versus late diagnosis on emotional response

**TABLE 6:** Themes with quotes.

Theme	Quotes
Fetal attachment	
During pregnancy	**Q1** “No, it still felt rather abstract, being pregnant. You don't really feel it [fetus] yet.” *R35, early TOP, mother*
During pregnancy	**Q2** “I already loved him, of course, because the moment you hold that pregnancy test in your hands, well, that's when your motherly feelings begin.” *R27, early TOP, mother*
During pregnancy	**Q3** “I could feel him very strongly, and I was very aware of him. I also felt very connected to him, as if we were truly experiencing it together. That bond kept growing, especially once I could feel him move.” *R48, late TOP, mother*
During pregnancy	**Q4** “I think that [attachment] came much more strongly later, when the baby started moving. Then we had the feeling that whenever I placed my hand on [name of the woman]'s belly, the baby would really move toward me. So, I think that created more of a bond than before.” *R48, late TOP, partner*
After the abnormal scan	**Q5** “I actually felt like, well, now she's still here, so I really wanted to experience it. I was really happy that I was able to feel that at that time.” *R2, late TOP, mother*
Parental identity after birth	**Q6** “Yes, I think, oh, that is our little girl. I think, she will always be our first little girl; she made us parents, yes.” *R49, early TOP, mother*
Parental identity after birth	**Q7** “[Regarding naming] No, not very deliberately. A practical reason because we didn't have a name ready yet. We still don't know to this day whether it was a boy or a girl.” *R45, early TOP, partner*
Parental identity after birth	**Q8** “I think, as many people often say, you do become parents, but you're left with empty hands. Normally, when you're a parent, you're busy, you have a child, you're happy, but now you're left arranging a funeral. It's obviously a very different experience from what you'd expect.” *R31, late TOP, mother*
Time pressure	**Q9** “No, absolutely not. Because I was also allowed to come back to the outpatient clinic for another ultrasound, they offered all kinds of things, and you could also go to [another tertiary care center] if you wanted to, and be referred. No, there really wasn't any pressure, especially because it was still so early in the pregnancy.” *R35, early TOP, mother*
	**Q10** “I mean, the results from the genetic testing came in at 23 + 0 weeks. Two days later, we had the last ultrasound and then 2 days after that, we had to make the decision. So, it felt like we only had a day and a half to decide.” *R18, late TOP, mother*
	**Q11** “I think I underestimated how serious the 13‐week scan could be and the consequences it might have. If I hadn't done the 13‐week scan and had just waited for the 20‐week scan, the diagnosis would've come as a huge shock—but everything would've happened very fast. There wouldn't have been that long period of waiting. But now, the prolonged uncertainty really weighed on me. That may have influenced my decision, because I didn't want to continue in that uncertainty. You don't have that with the 20‐week scan—you just immediately go for tests like an amniocentesis or CVS, and then you have to decide.” *R54, late TOP, mother*
Grief	**Q12** “I notice that life has just lost some of its shine. […] It feels different, it feels less deep.” *R18, late TOP, mother*
	**Q13** “I feel like I've almost lost—not that I'm truly depressed, but I've lost some of my sense of purpose in life. I also feel like our life has come to a standstill while everyone else's keeps moving forward.” *R15, early TOP, mother*
	**Q14** “We realized quite early in this process that we [partner and I] went through the same, but experienced it differently, I think that's the best way to describe it.” *R24, early TOP, mother*
Reflections on gestational age	**Q15** “So I think it's mainly the emotional connection you have with the child, that it's even stronger [at 20 weeks]. And now, well, I hadn't felt it move yet. I mean, I did feel that I was pregnant, physically, but I hadn't felt the child itself yet, it's all still very small. So I think that does make a difference. For me, it would have made a difference. So I was really relieved or, well, not exactly glad, but I did feel it was a good thing that the anomaly was found now, and not only at 20 weeks.” *R35, early TOP, mother*
	**Q16** “And I think, when I see what came out [at birth], what eh, how it was fitted out with everything, it was really just eh, a human being. And then I think, yes, how would it be at 20 weeks? So I think, both moments are difficult, but I would rather go through it earlier than at 20 weeks.” *R27, early TOP, mother*
	**Q17** “And if they had referred us earlier and we had still gotten this result with a chorionic villus sampling or something, I might have given birth at 15 weeks and that would have been really different from 23.5 weeks, I think. Especially because I could already feel him from 16 weeks. So, I had really 8 weeks, well, even [partner] felt him from the outside. And, yeah, you know it's a boy by then.” *R52 late TOP, mother*

### 3.10 Fetal attachment

#### 3.10.1 During pregnancy

About half of the women in the early TOP group described the absence of strong attachment to the fetus during pregnancy, citing reasons such as not feeling fetal movements or not having physical signs of pregnancy (Quote 1). However, the other half described having feelings of attachment, with some experiencing this from the moment of the positive pregnancy test (Quote 2). Most women with late terminations experienced fetal attachment during pregnancy, which was enhanced by fetal movements (Quote 3). Some partners in the late TOP group described a less concrete attachment early in pregnancy, with a perceived fetal connection shaped by future‐oriented dreams. As the pregnancy progressed, attachment increased, particularly with experiencing fetal movements (Quote 4).

#### 3.10.2 After the abnormal scan

In both groups, the abnormal ultrasound influenced fetal attachment for some participants—while some (*n* = 12 interviews) distanced themselves emotionally, others (*n* = 12 interviews) reported an even stronger bond and tried to embrace the remaining time (Quote 5).

#### 3.10.3 Parental identity after birth

Within the early TOP group, experiences of parental identity after birth varied, and no clear pattern emerged. Some (*n* = 11 interviews) felt a strong sense of becoming parents after birth, considering the child as a significant part of their lives (Quote 6). In contrast, others (*n* = 9 interviews) did not identify as parents, despite feeling love for the child. Around half of the participants chose to name the child, while the other half refrained, citing reasons such as it being too early, the gender being unknown, or that naming would make the situation feel too concrete (Quote 7). Most participants in the late TOP group described a strong sense of parental identity, though some expressed ambivalence (Quote 8). While they felt they had become parents, the experience was marked by a sense of loss and emptiness.

### 3.11 Time pressure

Participants in the early TOP group generally did not report time pressure associated with the legal limit for termination. Some attributed this to the early stage of pregnancy and the availability of supportive follow‐up options, which allowed them to feel they had sufficient time to consider their choice (Quote 9). However, for one couple, the advancing pregnancy and the prospect of feeling fetal movement created a different kind of time pressure, as they feared it would deepen their attachment and make the decision to terminate more difficult. In contrast, about half of the late TOP participants reported significant time pressure, often related to delays while waiting for genetic test results and the tight timeline for making a final decision (Quote 10). Several women also indicated that the pressure of time meant that a decision could not be postponed. However, others (*n* = 2 interviews) in the late TOP group did not report feeling pressured, as they made their decision quickly and therefore did not require additional time. One participant with a late TOP after an early referral due to abnormal FTAS results described the prolonged uncertainty about the prognosis as unbearable, rather than the process feeling rushed (Quote 11). Another participant with an abnormal FTAS and late TOP emphasized, however, that the FTAS resulted in more time for decision‐making, reducing any sense of time‐related urgency.

### 3.12 Grief

The majority of participants from both groups described ongoing emotional impact from the termination, regardless of gestational age (Quotes 12 and 13). A similar number of participants in both groups reported having been able to integrate the experience into their lives, allowing them to move forward to some extent. However, a larger proportion of participants in the late TOP group (*n* = 10 interviews vs. *n* = 4 interviews) expressed ongoing difficulties with moving forward. In both groups, differences between partners were noted in how grief was processed, with several partners internalizing their emotions. Some partners reported feeling disconnected due to the lack of a physical bond with the pregnancy, whereas others experienced guilt when reminded of the loss by their partner's grief. Despite these differences, participants acknowledged the depth of their grief and sought ways to support each other (Quote 14).

### 3.13 Reflections on gestational age

Many participants (*n* = 13 interviews) with an early termination expressed relief that the diagnosis was made after the FTAS, as they expected that a later diagnosis would have led to growing fetal attachment, more visible physical changes, and additional complexities in decision‐making (Quote 15 and 16). Some participants from the early TOP group found it challenging that people in their environment perceive an early loss as less significant, even though they felt that attachment had already started. Additionally, it was mentioned that the grief remains profound, regardless of the timing of the loss. In the late TOP group, similar reflections on gestational age were discussed (Quote 17).

## 4 DISCUSSION

This mixed method study evaluated the psychological impact and perspectives associated with early and late TOP. Questionnaire results showed that early TOP due to fetal anomalies is associated with lower psychological impact compared to late TOP. Although symptoms of anxiety, depression, distress and grief were present in both groups, women with late terminations more often reported clinically significant levels, particularly at 2 months postpartum. Furthermore, time to work resumption was longer in the late TOP group.

In‐depth interviews indicated that most late TOP participants reported strong fetal attachment, while experiences in the early TOP group were more varied. About half of the late TOP participants perceived time pressure in decision‐making due to the national legal limitation. TOP has a substantial emotional impact regardless of gestational age.

To our knowledge, this is the first large mixed methods study to explore the psychological impact and perspectives of an early versus late TOP due to fetal anomalies diagnosed by ultrasound. First‐trimester ultrasounds, such as dating and viability scans, have become increasingly common in routine obstetric care. These developments have enabled parents to visualize their fetus earlier and more vividly, potentially strengthening early attachment. This is supported by studies showing that fetal imaging techniques, such as 2D/3D/4D and virtual reality, can enhance maternal–fetal attachment.^28^, ^29^, ^30^ Earlier studies on the psychological impact of TOP, including those by Korenromp et al. performed in the Netherlands, did not distinguish between women with a known pretest risk of anomalies and those who were unexpectedly confronted with an ultrasound diagnosis. This may have led to important differences in psychological baseline and coping mechanisms that could influence the psychological impact of termination.

The difference in psychological impact observed between early and late TOP in our study may be explained by several factors. In the early group, a larger proportion of terminations were due to a lethal or serious anomaly with no available treatment, whereas in the late group, most terminations involved serious anomalies with available treatment, but with prognostic ambiguity. Previous research has showed higher levels of grief when fetal anomalies are presumed compatible with life.^4^ Moreover, a high degree of decisional doubt has been identified as a strong predictor of adverse psychological outcomes,^6^ which may be more likely in cases with nonlethal, but serious anomalies. Differences in the experience of fetal attachment may also contribute to variations in psychological impact between early and late TOP. Our interview data showed that participants in the early group reported a more varied experience of fetal attachment in pregnancy, while most participants in the late group described experiencing fetal attachment. Fetal attachment is known to increase with gestational age, especially after the onset of feeling fetal movements.^31^, ^32^ This was strongly supported by our interview data, in which both women and partners described how fetal movements and physical signs of pregnancy contributed to their feelings of attachment. Yet, some participants also reported experiencing attachment from the moment of a positive pregnancy test, underscoring the personal and subjective nature of fetal attachment.

Although differences in psychological impact and work resumption were observed between early and late TOP, both groups experienced substantial impact persisting at 6 months postpartum. Regardless of gestational age, the psychological impact of TOP for medical reasons remains profound and can be regarded as an inherent consequence of prenatal testing and the subsequent decision‐making process. This highlights the critical need for standardized psychological support to alleviate emotional distress and mitigate the broader psychosocial impact on bereaved parents. Moreover, providing comprehensive support to ensure that parents are able to make well‐informed, autonomous reproductive decisions is of paramount importance.^33^, ^34^ Future research should assess current care and identify parental needs to optimize support.

In our study, 22.5% of the questionnaire participants in the late TOP group indicated that they would have liked more time for decision‐making, compared to 2.7% in the early group. These findings were supported by the interviews, where approximately half of the late TOP participants reported experiencing time pressure due to the legal termination limit. In the Netherlands, pregnancy termination is legally allowed up to 24 weeks gestation.^35^ However, since the SAS is conducted between 18 and 21 weeks gestation, parents may have limited time for decision‐making. In contrast, the FTAS allows for earlier detection, potentially providing more time for consideration. Still, some participants described challenges related to earlier screening: one woman expressed a desire to decide on TOP before feeling fetal movements, while another found the extended period of diagnostic uncertainty emotionally distressing.

A strength of this study was the large sample size and the mixed methods design, which strengthened the reliability and depth of the findings and contributed to a broader understanding of the topic. The generalizability of the results was further enhanced by the inclusion of participants from various regions in the Netherlands. Additionally, conducting interviews online helped lower the threshold for participation.

Several limitations should be considered when interpreting the findings. Most participants were Dutch and highly educated, which may limit generalizability. Characteristics of nonresponders were not available and the number of eligible women who declined participation was not available, although the questionnaire response rate was high. Few interviews were conducted with participants with a late TOP following an abnormal FTAS, which is an exceptional long diagnostic trajectory that may influence their experience. Furthermore, baseline measure of mental well‐being was not known, making it difficult to account for this in the analysis. Part of the reported fetal anomalies were self‐reported, which may affect accuracy, although most diagnoses could be classified. Questionnaire time points were chosen to assess both short‐ and longer‐term psychological impact of TOP, while interviews were scheduled to maximize response rates and allow inclusion of participants from the questionnaire study. Finally, we were unable to assess psychological outcomes based on mode of delivery, although dilation and evacuation after an abnormal FTAS is not common practice in the Netherlands.

## 5 CONCLUSION

Our findings suggest that early TOP is associated with lower psychological impact compared to late TOP, mainly in the first months after delivery. This may be attributed to less intense fetal attachment and more time for reproductive decision‐making for some parents, supporting the presumed benefit of earlier intervention. Nevertheless, TOP carries a significant emotional impact at any gestational age.

## AUTHOR CONTRIBUTIONS

Conceptualization/methodology: EERL, MNB, KB, MCH, LH, NMTHC, CMB, RJHG, ES. Investigation (Data collection): EERL, KB. Formal Analysis: EERL, MNB. Data Curation: EERL, KB. Project Administration: EERL, MNB, KB, MCH. Supervision: MNB. Writing—Original Draft: EERL, MNB. Writing—Review and Editing: All authors. Visualization: EERL. Validation (Interpretation of data): All authors.

## FUNDING INFORMATION

The IMITAS study is supported by a grant from The Netherlands Organization for Health Research and Development (ZonMw, No. 543010001). The funding source was not involved in the study design; the collection, analysis, or interpretation of data; the writing of the report; or the decision to submit the article for publication.

## CONFLICT OF INTEREST STATEMENT

None declared.

## ETHICS STATEMENT

Ethical approval was granted by the Dutch Ministry of Health, Welfare, and Sport (license 3 219 987‐1 012 059‐PG) and based on positive advice by the Health Council of the Netherlands (No. 2021/30, June 30, 2021). The phase before FTAS introduction was approved by the Medical Ethical Review Committee Utrecht (No. 20/765, December 1, 2020). Written informed consent was obtained from all participants.

## Supporting information


**Appendix S1.** Interview guide.

**Table S1.** Subanalysis on psychometric outcomes for women with a late TOP.

## Data Availability

The data that support the findings of this study are available from the corresponding author upon reasonable request.
