# Supplementary material for: Early versus late termination for fetal anomalies: Women's perspectives and psychological impact in a mixed methods study
Source: Acta Obstet Gynecol Scand. 2026 Jan 9;105(3):444–54. doi: 10.1111/aogs.70122 (PMC12942052; doi:10.1111/aogs.70122)
Supplement: Supplementary file 1 — Appendix S1. Interview guide. [file AOGS-105-444-s001.docx]

**Appendix S1:** Interview guide

The aim of this interview is to assess how women (and their partners, if applicable) have experienced pregnancy termination due to fetal anomalies. Participants will be invited to share their decision-making process and to reflect on the impact of the abnormal scan and termination. They will be encouraged to express their perspectives, emotions and opinions on what they consider important. Participants will be encouraged to speak freely, while the interviewer ensures that key themes relevant to the study are addressed in the course of the discussion.

Before recording, participants are welcomed and introduced to the study objectives by the interviewer. They will be informed that participation is voluntary and that the interview will be recorded (with consent). It will be emphasized that participating is voluntary and arrangements of confidentiality will be set out. Participants will be reminded that there are no right or wrong answers and that the interviewer is interested in their individual perspectives in their own words. They will also be informed that they are free to decline to answer any questions or discuss any topics they prefer not to.

The actual interview will start then with an open question: “How was the first period of your pregnancy? What was it like to be pregnant?”. Respondents will be encouraged to share their thoughts, views and feelings. The interviewer will then use follow-up questions to explore key themes from the interview guide. The interview will be finished by inviting the participant to share any final thoughts or comments they may have regarding the interview. Following this, the participant will be sincerely thanked for their valuable contribution. Contact details of the researchers will be provided for any further questions.

**Topics**

1. Decision-making process
2. Perception of the future child (fetal attachment, parental identity)
3. Mental well-being
4. Grief
5. Coping
6. Support (during decision-making, after pregnancy termination)

| Interview identification code | …………. |
| --- | --- |
| Date of interview | …………. |
| Age (also from partner, if applicable): | …………. |
| Gravidity | …………. |
| Parity | …………. |
| Number of living children | …………. |
| Highest level of education (also from partner, if applicable): | …………. |
| Country of origin: | …………. |
| Country of origin parents: | …………. |
| Living situation | □ Living with / married to partner □ In a relationship but not living together □ Single □ Other |
