# Supplementary material for: Early versus late termination for fetal anomalies: Women's perspectives and psychological impact in a mixed methods study
Source: Acta Obstet Gynecol Scand. 2026 Jan 9;105(3):444–54. doi: 10.1111/aogs.70122 (PMC12942052; doi:10.1111/aogs.70122)
Supplement: Supplementary file 2 — Table S1. Subanalysis on psychometric outcomes for women with a late TOP. [file AOGS-105-444-s002.docx]

**Table S1:** Subanalysis on psychometric outcomes for women with a late TOP.

| Outcome measures | Abnormal FTAS*  (*n*=20) | Abnormal SAS with normal FTAS^†^ (*n*=75) | Abnormal SAS with no FTAS^‡^ (*n*=34) | P |
| --- | --- | --- | --- | --- |
| Anxiety (STAI) |  |  |  |  |
| *2 months postpartum (T1)* |  |  |  |  |
| Mean ± SD | 45.2±11.4 | 46.4±12.3 | 46.0±9.4 | 0.918 |
| Clinically significant score (≥40,   n (%)) | 13 (65.0) | 55 (73.3) | 26 (76.5) | 0.651 |
| *6 months postpartum (T2)* |  |  |  |  |
| Mean ± SD | 40.6±8.7 | 43.8±11.9 | 43.9±12.3 | 0.550 |
| Clinically significant score (≥40, n   (%)) | 11 (61.1) | 39 (52.0) | 22 (66.7) | 0.907 |
|  |  |  |  |  |
| Depression (EDS) |  |  |  |  |
| *2 months postpartum (T1)* |  |  |  |  |
| Median IQR | 5.0 (3.3-7.8) | 5.0 (3.0-8.0) | 6.0 (3.0-8.0) | 0.980 |
| Moderate symptom level (≥7, n   (%)) | 7 (35.0) | 28 (37.3) | 14 (41.2) | 0.889 |
| *6 months postpartum (T2)* |  |  |  |  |
| Median IQR | 4.0 (0.8-6.3) | 4.0 (2.0-7.0) | 4.0 (1.0-7.0) | 0.777 |
| Moderate symptom level (≥7, n   (%)) | 4 (22.2) | 17 (27.4) | 9 (27.3) | 0.902 |
|  |  |  |  |  |
| Post-traumatic stress (IES) |  |  |  |  |
| *2 months postpartum (T1)* |  |  |  |  |
| Mean ± SD | 29.9±14.2 | 33.7±13.4 | 36.4±12.3 | 0.227 |
| Clinically significant score (≥26, n   (%)) | 11 (55.0) | 54 (72.0) | 28 (82.4) | 0.096 |
| Moderate impact (26-43, n (%)) | 8 (40.0) | 38 (50.7) | 20 (58.8) | 0.406 |
| Severe impact (≥44, n (%)) | 3 (15.0) | 16 (21.3) | 8 (23.5) | 0.752 |
| *6 months postpartum (T2)* |  |  |  |  |
| Mean ± SD | 26.3±15.0 | 28.3±12.2 | 33.2±13.8 | 0.133 |
| Clinically significant score (≥26, n   (%)) | 10 (55.6) | 36 (58.1) | 24 (72.7) | 0.311 |
| Moderate impact (26-43, n (%)) | 8 (44.4) | 28 (45.2) | 16 (48.5) | 0.943 |
| Severe impact (≥44, n (%)) | 2 (11.1) | 8 (12.9) | 8 (24.2) | 0.295 |
|  |  |  |  |  |
| Grief (PGS) |  |  |  |  |
| *2 months postpartum (T1)* |  |  |  |  |
| Mean ± SD | 85.2±17.3 | 86.7±23.1 | 84.7±18.4 | 0.888 |
| Clinically significant score (≥91, n   (%)) | 9 (45.0) | 30 (40.0) | 13 (38.2) | 0.884 |
| *6 months postpartum (T2)* |  |  |  |  |
| Mean ± SD | 82.3±20.4 | 82.0±23.4 | 79.5±23.7 | 0.866 |
| Clinically significant score (≥91, n   (%)) | 6 (33.3) | 23 (37.1) | 9 (27.3) | 0.627 |
| Abbreviations: EDS, Edinburgh Depression Scale; IES, The Impact of Event Scale; PGS, Perinatal Grief Scale; SD, Standard deviation; STAI, State-Trait Anxiety Inventory; TOP, Termination of pregnancy.  *Six months postpartum, abnormal FTAS *n=*18  †Six months postpartum, abnormal SAS with normal FTAS *n=*62  ‡Six months postpartum, abnormal SAS with no FTAS *n=*33 | | | | |
